# Radiofrequency Electromagnetic Field Exposure and the Resting EEG: Exploring the Thermal Mechanism Hypothesis

**DOI:** 10.3390/ijerph16091505

**Published:** 2019-04-28

**Authors:** Sarah P. Loughran, Adam Verrender, Anna Dalecki, Catriona A. Burdon, Kyoko Tagami, Joonhee Park, Nigel A. S. Taylor, Rodney J. Croft

**Affiliations:** 1 Australian Centre for Electromagnetic Bioeffects Research (ACEBR), Illawarra Health and Medical Research Institute, School of Psychology, University of Wollongong, Northfields Ave, Wollongong, NSW 2522, Australia; adamv@uow.edu.au (A.V.); adalecki@uow.edu.au (A.D.); nigelastaylor@gmail.com (N.A.S.T.); rcroft@uow.edu.au (R.J.C.); 2 Centre for Population Health Research on Electromagnetic Energy (PRESEE), School of Public Health and Preventive Medicine, Monash University, Melbourne, VIC 3004, Australia; 3 Centre for Human and Applied Physiology, School of Medicine, University of Wollongong, Wollongong, NSW 2522, Australia; cburdon@uow.edu.au (C.A.B.); tagami.kyouko@kao.co.jp (K.T.); jh1811@snu.ac.kr (J.P.)

**Keywords:** radiofrequency electromagnetic fields, body temperature, electroencephalogram, mobile phones, exposure limits

## Abstract

There is now strong evidence that radiofrequency electromagnetic field (RF-EMF) exposure influences the human electroencephalogram (EEG). While effects on the alpha band of the resting EEG have been repeatedly shown, the mechanisms underlying that effect have not been established. The current study used well-controlled methods to assess the RF-EMF exposure effect on the EEG and determine whether that effect might be thermally mediated. Thirty-six healthy adults participated in a randomized, double-blind, counterbalanced provocation study. A water-perfusion suit (34 °C) was worn throughout the study to negate environmental influences and stabilize skin temperature. Participants attended the laboratory on four occasions, the first being a calibration session and the three subsequent ones being exposure sessions. During each exposure session, EEG and skin temperature (8 sites) were recorded continuously during a baseline phase, and then during a 30 min exposure to a 920 MHz GSM-like signal (Sham, Low RF-EMF (1 W/kg) and High RF-EMF (2 W/kg)). Alpha EEG activity did not change during either of the exposure conditions compared to the Sham condition. As a measure of thermoregulatory activation, finger temperature was found to be higher during both exposure conditions compared to the Sham condition, indicating for the first time that RF-EMF exposure studies cause thermoregulatory changes. This supports the feasibility of the hypothesis that RF-EMF effects on alpha EEG activity are mediated via a thermal mechanism.

## 1. Introduction

Although previous research has largely been unable to identify acute effects of radiofrequency electromagnetic field (RF-EMF) exposure on humans, an important exception is the effect on the resting electroencephalogram (EEG), where the ‘alpha’ frequency range has been consistently affected, with the majority of the methodologically appropriate studies (i.e., those that used eyes open EEG to avoid ceiling effects) showing an increase in alpha activity (e.g., [1,2,3]). Similar effects have also been shown in the ‘sleep spindle’ frequency range of the sleep EEG (e.g., [4,5,6,7,8,9]). While these effects have been replicated repeatedly and in independent laboratories, clinically relevant consequences of these changes, such as changes in cognitive performance, as well as the mechanisms underlying the effect on the EEG, have not been established [10,11].

Despite no evidence relating these impacts of RF-EMF exposure to health [10,11], the lack of an established mechanism for the effects seen on the EEG remains problematic. One suggestion is that the presence of modulations in the RF-EMF signal, such as those used by the Global System for Mobile Communications (GSM), may be an important factor behind such biological effects [12]. Indeed, most of the studies to date reporting effects on the alpha band of the EEG have used pulse-modulated RF-EMF exposures. The importance of pulse-modulation for inducing effects was also supported by Huber et al. [13], who reported that a pulse-modulated RF-EMF exposure applied at 1 W/kg had an impact on the EEG, whereas applying a continuous wave (i.e., without modulation) at the same level of 1 W/kg did not induce an effect on the EEG. They concluded that pulse-modulation of RF-EMF is crucial to induce changes in brain physiology, and that this also indicated that the mechanism underlying such changes could not be of a thermal origin [13]. However, this argument remains problematic for a number of reasons. Firstly, it cannot be ruled out that small temperature variations within normal physiological fluctuations are capable of altering the EEG. Secondly, neurons respond differently to temperature gradients [14]. The studies comparing pulse-modulated and continuous wave RF-EMF exposures (e.g., [13]) were not equal in terms of peak power and temperature profile over time, which is important, because temperature profiles will affect cell-firing patterns [14]. Indeed, we recently demonstrated that when peak power was kept equivalent between pulse-modulated and continuous wave RF-EMF exposures, the effect on the EEG did not differ between continuous and pulse-modulated RF-EMF [15]. Based on that outcome it can be concluded that a non-thermal mechanism has not been identified and that a thermal mechanism remains a possibility as the mechanism underlying the effects of RF-EMF exposures on the EEG. Similarly, a thermal mechanism has also been suggested as potentially being responsible for the effects of RF-EMF exposure on the sleep EEG [16].

An underlying thermal mechanism, however, presents problems which still remain to be addressed. For instance, the magnitude of energy change, and the corresponding elevation in tissue temperature from RF-EMF exposures associated with telecommunications technology is small (<0.1 °C), especially when compared to circadian (0.8–1.0 °C; [17]) or exercise-induced temperature variations (>1.0 °C; [18]). Such a small temperature increase is too small to induce any kind of cell damage and is unlikely to elicit corrective autonomic responses. Thus, it is thought that a thermal mechanism could not be responsible for the observed EEG changes. However, previous studies have not actually tested the possibility that the central nervous system can in fact detect and initiate responses to such small temperature changes in a physiologically meaningful way, particularly if they occur within the most thermally sensitive tissues [19].

The purpose of the current study was to determine whether the human central nervous system can indeed detect small temperature changes associated with RF-EMF exposures within the current international exposure limits, as designated by the International Commission on Non-Ionizing Radiation Protection (ICNIRP; [20]). One particularly effective way of doing this is to determine whether such temperature changes are physiologically sufficient to engage meaningful thermoregulatory processes. Therefore, the current study was designed using a number of methodological improvements, including the thermal clamping of the non-exposed skin temperatures, with the aim of not only replicating the effect of RF-EMF exposure on the alpha band of the awake EEG but also determining, for the first time, whether that response is accompanied by physiologically meaningful thermal processes.

## 2. Materials and Methods

### 2.1. Participants

Thirty-six right-handed, healthy volunteers (18 males and 18 females) aged between 18 and 52 years (mean age = 24.4 years; standard deviation = 6.3 years) participated in the current study. Participants with a history of seizures, epilepsy, or serious head injury or taking any neuroleptic medication were excluded. All participants were required to abstain from consuming alcohol for 8 hours and from caffeinated foods and beverages for 1 hour prior to arrival at the laboratory, as these substances affect central nervous system activity and may confound the experimental manipulation. Use of mobile phones to make or receive a phone call was also prohibited for 2 hours prior to arrival at the laboratory. The University of Wollongong Human Research Ethics Committee approved the study protocol (HE:13/146), with written, informed consent obtained from all volunteers prior to participation.

### 2.2. Study Design

A randomized double-blind, counterbalanced, crossover design was used in which participants were required to attend four laboratory sessions, with each session separated by at least seven days. Following a preliminary calibration session (to minimize practice effects on cognitive task performance and determine individually appropriate difficulty levels, see [21]), each participant underwent three subsequent sessions, each involving a different exposure condition (Sham: 0 W/kg, Low RF: 1 W/kg, and High RF: 2 W/kg). The exposure condition order was randomized and fully counterbalanced across participants and within each gender.

### 2.3. Physiological Measurements and Apparatus

#### 2.3.1. EEG Recordings

Participants were fitted with a 19-channel EEG cap (Quik-Cap, Compumedics, Neuroscan, Abbotsford, Victoria, Australia), and EEG data were recorded from the FP1, FP2, Fz, F3, F4, F7, F8, Cz, C3, C4, T7, T8, Pz, P3, P4, P7, P8, O1, O2, and M2 electrode locations according to the international 10/20 system. All EEG data were referenced to the left mastoid (M1) and grounded midway between FPz and Fz. Electro-oculogram (EOG) data were recorded from above and below the left eye and from the outer canthus of the left and right eyes. All EEG and EOG data were recorded with an online 0.05–500 Hz analogue band-pass filter and a sample rate of 2000 Hz. All electrode impedances were below 5 kΩ at the start of each recording. The EEG amplifier was placed inside an RF-shielded box, and as has been shown previously, effects of exposure on electrodes are minimal [22].

#### 2.3.2. Skin Temperatures, Body Core Temperature, and Mean Arterial Pressure

**Figure 1:**
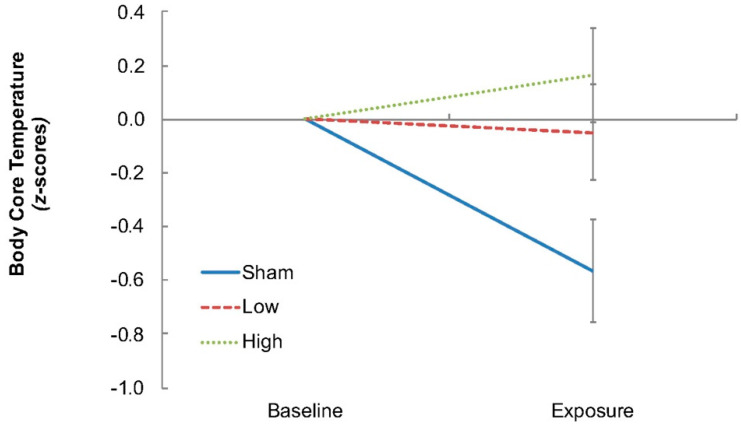
Body core temperatures across exposure conditions, as measured from the auditory canal. As shown here, temperature increased immediately in both exposure conditions relative to the Sham condition (exposure measurement corresponds to the average of the first 4 min of exposure), which due to thermal inertia is too early to represent a real change in body core temperature. Furthermore, this change does not reflect the lack of body core temperature change due to much larger whole body radiofrequency exposures [24], nor the longer (22–34 min) body core thermal time constants modelled using radiofrequency exposure conditions more conducive to body core temperature elevation (e.g., [25]).

To estimate the whole-body thermal energy content of each participant, temperature measurements from both the skin and deep-body (core) tissues were recorded [18]. Because skin temperatures are widely variable across sites under temperate conditions [18], it is necessary to take measurements from multiple sites. Furthermore, since this experiment was dependent upon whole-body thermal clamping, then it was necessary to confirm the veracity of the thermal clamp. Accordingly, eight skin temperatures were simultaneously measured (forehead, right chest, right scapula, right upper arm, right forearm, left dorsal hand, right anterior thigh and right calf), using thermistors attached to the skin with a single layer of waterproof tape (YSI type-EU, Yellow Springs Instruments, Yellow Springs, OH, USA). In addition, the skin temperature from the dorsal surface of the distal phalanx of the left middle finger was measured to reflect variations in local skin blood flow [23]. In experiments such as this, when routine physiological measurements are not possible, surrogate indices become necessary, and finger temperature was one such index. A separate thermistor was used to record ambient temperature. All temperatures were sampled continuously throughout the experimental sessions (15 s intervals; 1206 Series Squirrel, Grant Instruments Ltd., Shepreth, Cambridgeshire, UK). Body core temperature (auditory canal) was also recorded, however, due to the proximity of the RF-EMF exposure device to the auditory canal, that index, in effect, operated as a local temperature measurement and was found to no longer track changes in body core temperature (Figure 1). Since the use of esophageal temperature was impractical for this population sample for the same reasons, and since gastrointestinal and rectal temperatures are relatively insensitive conduction-dependent indices [18], those alternatives were also inappropriate and not used. The recording of body core temperature under these circumstances remains problematic and awaits resolution. Systolic and diastolic blood pressures were recorded during baseline and at the beginning and end of each exposure period (Omron SEM-2, Omron Healthcare Inc., Kyoto, Japan).

#### 2.3.3. Water-Perfusion Suit

To minimize the impact of ambient temperature and to stabilize skin and body core temperatures, thermal clamping was used. This was achieved using a full-body, water-perfusion suit that was fitted at the start of each experimental session (Cool Tubesuit, Med-Eng, Ottawa, ON, Canada). The suit contained in-series plastic tubes which were secured to the inside of an elasticized, whole-body garment. The suit was then connected to a water bath and pump which distributed water through the tubes at a rate of 2.5 L/min throughout the experiment (38 L water bath; Type VFP, Grant Instruments, Cambridge, UK). The water temperature was regulated at 34 °C and perfused the suit for a minimum of 10 min before experimental testing began. As fitted for this experiment, the suit covered the torso, the arms to the wrist level, and the legs to the level of the ankles. Therefore, with the exception of the forehead, hand, and finger, the majority of the skin temperature sites were contained beneath the suit. As the sensors were in intimate contact with the skin and separated from the perfusion suit by a layer of tape and variable pockets of air, they responded primarily to changes in skin temperature, which were effectively clamped by the perfusion suit.

### 2.4. Radiofrequency Exposure

Each participant underwent three exposure conditions using an sXh920 planar exposure system, which generated a 920 MHz GSM-like signal (IT’IS Foundation, Zurich, Switzerland). The exposure system was calibrated to provide a peak spatial specific absorption rate (SAR) averaged over 10 g of tissue of 0 W/kg (Sham), 1 W/kg (Low RF), and 2 W/kg (High RF). These were the levels of the applied exposures in each condition, and given that exposures were applied in a Faraday cage with 80 dB attenuation, any additional RF-EMF from stray fields would be minimal. These exposures all complied with the international exposure limits, as designated by ICNIRP [20]. An RF antenna was placed in a box mounted on a wooden pillar, with the participants seated in between this and another box (identical to the box housing the antenna, but not containing an antenna). The antenna was positioned on the left side of each participant’s head at a distance of 115 mm, with the center of the antenna positioned 42 mm above the auditory canal, ensuring a consistent and well-defined position throughout. Double-blinding was achieved by having the exposure system and conditions programmed by an independent researcher, and through the use of brown noise throughout the experiment to eliminate acoustic perception of the exposure. This was also subjectively verified at the completion of each experimental testing session, and as described in Verrender et al. [21], no participant was able to correctly identify all exposure conditions, indicating that blinding was maintained throughout.

### 2.5. Procedures

Participants arrived at the laboratory at either 09:00 or 13:00 for all experimental sessions. To eliminate circadian influences, time of participation was kept consistent within participants. At the beginning of each session, participants completed a 16-item visual analogue mood scale [26], and a short questionnaire pertaining to their prior sleep, caffeine and alcohol consumption, and mobile phone use. Participants were then fitted with the EEG and other physiological apparatus, as described above, as well as the water-perfusion suit, before being seated comfortably inside a Faraday cage facing a computer screen (at a distance of approximately 90 cm) and with the head positioned in between the two boxes. At this point, the water pump was activated to allow for a normothermic baseline state to be reached before any experimental testing began. During this time, participants completed practice versions of the cognitive tasks (results published previously in [21,27] and not presented further here).

At the conclusion of the set-up and practice tasks, participants completed a 16 min ‘Baseline’ block, during which they were not exposed to RF-EMF. During this time, participants underwent an EOG correction task [28], and resting EEG and physiological data were also recorded. This was followed by two 30 min experimental blocks, with the first block always being the exposure block (Sham, Low, or High RF-EMF, depending on counterbalancing) and the second block identical to the first, except that exposure was always off. The two blocks were separated by a 1 min rest period. At the beginning of the experimental blocks, resting EEG (min 0–4) and mean arterial pressures were recorded. Participants then completed a 16 min cognitive test battery (see [21]), and resting EEG (min 22–26) and mean arterial pressure data were again recorded at the end of the block. Upon completion of the experimental testing blocks, all monitoring equipment was removed, and participants completed another 16-item visual analogue mood scale and an exposure questionnaire to assess awareness of variations in the exposure conditions.

### 2.6. Data Analysis

Resting, eyes-open EEG data were down-sampled to 500 Hz (125Hz low-pass filter, 12dB/oct), re-referenced to digitally linked mastoids, EOG corrected [28], and epoched into 2 s segments. Epochs were spline-fit to 512 points, baseline corrected, and artifact rejected (±100 µV at any scalp electrode site; mean epochs rejected = 7.5%, SD = 12.3%). The remaining artifact-free epochs were averaged in the frequency domain (Fast Fourier transform; Hanning window; 20%), with alpha power (8–12 Hz) calculated for nine electrode sites (Frontal: Fz, F3, F4; Central: Cz, C3, C4; Parietal: Pz, P3, and P4).

An area-weighted summation of the eight skin temperatures was used to derive mean skin temperature [29]. Mean skin temperatures were then calculated as an average over 4 min segments from Baseline and the Exposure Block to verify the success of the thermal clamping procedure (Baseline; Time 1 = 0–4 min; Time 2 = 8–12 min; Time 3 = 15–19 min; Time 4 = 23–27 min). Mean arterial pressure was derived as systolic pressure plus 0.33 times the difference between the systolic and diastolic pressures.

### 2.7. Statistical Analyses

Statistical analyses were performed using SPSS (Version 23, IBM Corporation, Armonk, NY, USA), with all data converted to *z*-scores. This conversion normalizes the data, ultimately reducing variance and therefore providing a more effective way of detecting a difference, if one exists. All *z*-scores were calculated by: (1) calculating the mean and standard deviation of each measurement for each participant for each time period of interest (e.g., baseline, exposure time point); then (2) taking the raw data values, subtracting the mean and dividing by the standard deviation described above in (1). Initially, a 3 (Condition: Sham, Low, High) × 5 (Time: Baseline, Time 1, Time 2, Time 3, Time 4) repeated measures ANOVA was performed on the mean skin temperature data to verify thermal clamping.

Subsequently, to verify previously reported effects of RF-EMF exposure on EEG alpha activity, a 3 (Condition: Sham, Low, High) × 3 (sagittality: frontal, central, parietal) × 2 (laterality: left, right) repeated measures ANOVA was performed. The inclusion of sagittality and laterality measures provides for the most appropriate account of error variance; however, as they did not specifically relate to the hypotheses, they were not analyzed further. Therefore, only planned contrasts (one-tailed) testing whether EEG alpha power was higher during each of the exposure conditions compared to the Sham condition were performed at time point 4 (23–27 min; Sham-versus-Low RF-EMF, and Sham-versus-High RF-EMF).

For finger temperature, a repeated measures ANOVA (Factor: Condition) was performed, with planned contrasts (one-tailed) to compare the Sham-versus-Low and the Sham-versus-High RF-EMF exposures at the end of the 30 min exposure period to determine whether finger temperature increased relative to the Sham condition. A repeated measures ANOVA (Factor: Condition) was also performed on mean arterial pressure and air temperature measures at time point 4 (23–27 min), with the latter aimed at verifying that no differences existed across conditions. 

## 3. Results

### 3.1. Verification of Thermal Clamping

Ambient temperature increased across the course of the experiment, which was expected due to the presence of several significant heat sources (participant plus equipment) within a relatively small space (the Faraday cage). However, no significant differences were seen between the exposure conditions (*p* = 0.83; see Table 1). Furthermore, mean skin temperature did not differ between the three exposure conditions (*p* = 0.33; see Table 1), confirming that thermal clamping was effective at maintaining skin temperature constant throughout the experiment, and thus eliminating the impact of ambient temperature influences. 

### 3.2. Effect of RF-EMF Exposure on the EEG

**Figure 2:**
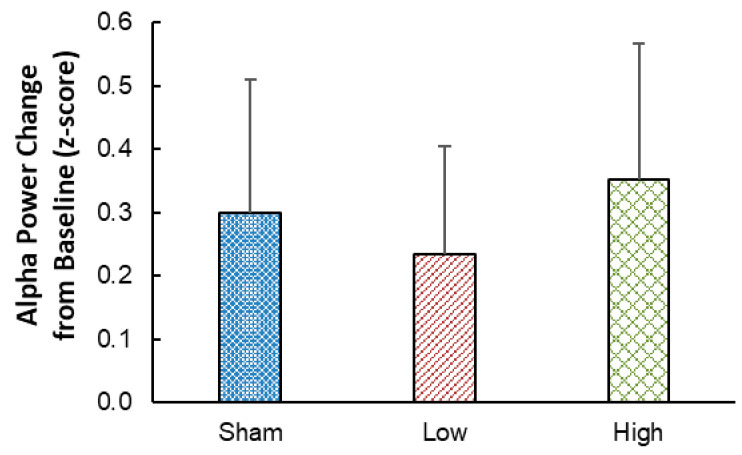
The change in EEG alpha power between Baseline and the end of exposure (min 22–26) in each of the Sham, Low, and High RF-EMF exposure conditions. Error bars denote standard errors of the means.

Resting EEG alpha power did not differ between the High RF-EMF exposure condition compared to the Sham condition (*p* = 0.43), and no difference was found between the Sham and Low RF-EMF exposure conditions (*p* = 0.40; see Figure 2 and Table 2).

### 3.3. Effect of RF-EMF Exposure on Finger Temperature

**Figure 3:**
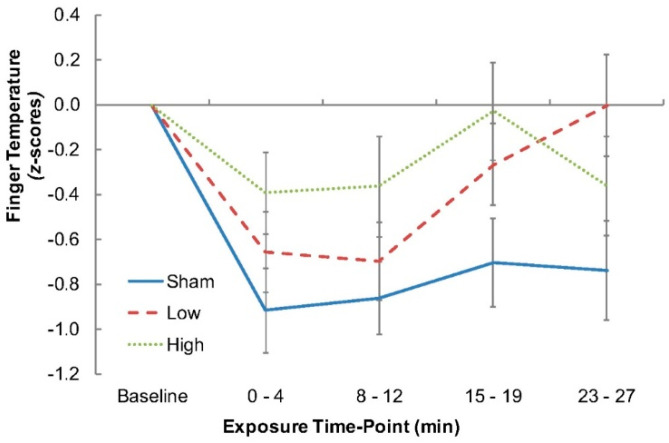
Mean change in finger temperature from Baseline to the end of exposure for the Sham, Low, and High exposure conditions (error bars denote standard errors of the means).

Finger temperature was higher during the Low RF-EMF exposure condition compared to the Sham condition (*p* = 0.01), and this was also the case for the High RF-EMF exposure condition compared to the Sham condition (*p* = 0.09), although the latter difference only reached trend-level. Changes in finger temperature across the experiment are shown in Figure 3 (for raw data see Table 2).

### 3.4. Effect of RF-EMF Exposure on Mean Arterial Pressure

No difference was seen between the Low RF-EMF exposure and Sham exposure conditions for mean arterial pressure (*p* = 0.57), while a trend-level decrease in mean arterial pressure for the High RF-EMF exposure condition compared to the Sham exposure condition was seen (*p* = 0.09; Table 3).

## 4. Discussion

Employing a tightly controlled methodology, the present study was designed to extend previous reports of RF-EMF exposure-related enhancements to the alpha band of the EEG and to determine, for the first time, the possibility that these alpha changes may be related to thermal effects on the central nervous system. The results did not replicate previous evidence for an impact of RF-EMF exposure on the alpha band of the EEG. However, the study demonstrated an increase in finger temperature in the exposure conditions relative to the Sham condition, which shows that the small but highly specific and localized thermal change caused by the RF-EMF exposure was sufficient to engage thermoregulatory processes.

Failing to replicate the majority of previous research, there was no increase in EEG alpha activity during RF-EMF exposure when compared to the Sham condition. Although previous studies have shown an impact on the EEG at lower exposure levels, similar to the 1 W/kg employed in our Low exposure condition (e.g., [5,30]), as well as some indication of a possible dose–response relationship (e.g., [31]), neither of those effects were observed in the current study. Furthermore, as detailed in Verrender et al. [21], no clear functional consequences of this change in EEG alpha activity were shown, providing further evidence that small changes in EEG activity do not have important functional consequences.

Novel to this experiment was the use of numerous physiological techniques to more tightly control (clamp) the heat content of the cutaneous tissues [32], and thereby remove artifactual thermal influences and stabilize skin temperatures. Importantly, all of the measures used were shown to be effective, verifying the utility of these methods. This is particularly important as the temperature changes accompanying RF-EMF-induced molecular oscillations are far smaller than those associated with local ambient influences. By minimizing those thermal ‘artifacts’, we were able to provide an insight into the mechanism underlying previously reported EEG alpha effects by tracking thermal physiological responses.

Despite the presence of whole-body clamping, finger temperatures were consistently higher during each of the RF-EMF exposures relative to the Sham condition. Since cutaneous blood flow to the hands and feet is exquisitely sensitive to changes in heat storage [33], the effect of which is most powerfully driven by thermosensitive neurons within the central nervous system [19], this would suggest that the observed increases in finger temperature, as a surrogate for local blood flow, reflected a physiological response (cutaneous vasodilation of the hands) to the RF-EMF-induced heating of the central thermosensitive tissues. The gradual reduction in finger temperature observed during each condition would be expected, since the participants remained stationary for an extended duration. Thus, the effect of RF-EMF-induced heating was to reduce the rate of that decline. As a consequence, this investigation may be the first to show a thermal mechanism that might underly the previously reported EEG alpha change.

Aside from the ramifications of the changes in finger temperature, the time course of those changes provides an interesting insight into how the effect might occur and in relation to the dose response of the effect. Specifically, the time course of the finger temperature response differed between the two exposure conditions, with an immediate thermoregulatory response observed in the High exposure condition and a weaker, more protracted effect seen in the Low exposure condition. Both exposures showed a similar effect on temperature by the end of exposure. This is an important consideration because, if the EEG effect is a result of thermal changes, then it would be expected that the EEG changes would also follow a similar time course, which is something that has not previously been taken into consideration within the RF-EMF EEG studies reporting on dose–response effects (e.g., [31]).

There are several important factors that require further consideration, both in the current study and for future studies. Firstly, since finger temperature is a surrogate for the local vascular responses, at least in the presence of thermal clamping, then it would seem that these RF-EMF exposures resulted in relatively inconsequential, but physiologically effective, increases in the temperature of the central thermosensitive neurons, the result of which appeared to be cutaneous vasodilatation. This underscores the importance of precisely regulating the thermal environment. Secondly, although the finger temperature data were consistent with a change in local blood flow, that observation requires verification. Thirdly, if the change in EEG alpha is a result of thermal changes as the present study might indicate, then equivalent temperature loads from non-RF-EMF sources should induce similar effects, although this is yet to be determined. Finally, our results imply that different levels of exposure can indeed result in different temporal patterns of thermal response. This has not previously been considered, and if EEG alpha changes are a result of a thermal response, this may help to explain the differences in effects reported by earlier studies, including less than reliable dose–response relations.

## 5. Conclusions

The present study did not replicate previous evidence that the alpha band of the resting EEG is enhanced by RF-EMF exposures, commensurate with those emitted by modern telecommunications technologies. However, this is the first study to show that RF-EMF exposures are sufficient to engage a thermoregulatory response, and therefore it is consistent with an underlying thermal mechanism being responsible for the changes observed in brain physiology. 

## Figures and Tables

**Table 1:** Mean ambient temperature and mean skin temperature at Baseline and throughout exposure for the Sham, Low, and High exposure conditions (standard errors in parentheses).

**Time Point (min)**	**Sham**				
	**Baseline**	**0–4**	**8–12**	**15–19**	**23–27**
Mean Skin Temperature (°C)	35.88 (0.33)	35.88 (0.33)	35.84 (0.32)	35.83 (0.32)	35.82 (0.32)
Air Temperature (°C)	22.56 (1.27)	22.63 (1.28)	22.70 (1.29)	22.75 (1.30)	22.77 (1.30)
**Time Point (min)**	**Low**				
	**Baseline**	**0–4**	**8–12**	**15–19**	**23–27**
Mean Skin Temperature (°C)	35.80 (0.30)	35.80 (0.30)	35.80 (0.29)	35.80 (0.29)	35.80 (0.30)
Air Temperature (°C)	22.49 (1.01)	22.59 (1.02)	22.65 (1.02)	22.71 (1.02)	22.74 (1.00)
**Time Point (min)**	**High**				
	**Baseline**	**0–4**	**8–12**	**15–19**	**23–27**
Mean Skin Temperature (°C)	35.85 (0.32)	35.85 (0.32)	35.85 (0.32)	35.85 (0.31)	35.84 (0.30)
Air Temperature (°C)	22.51 (1.32)	22.58 (1.36)	22.65 (1.37)	22.70 (1.38)	22.70 (1.36)

**Table 2:** EEG alpha and finger temperature values at Baseline and end of RF-EMF exposure for the Sham, Low, and High Exposure conditions (standard errors in parentheses).

Time Point (min)	Sham		Low		High	
	Baseline	23–27	Baseline	23–27	Baseline	23–27
EEG Alpha (μV^2^)	1.75 (0.05)	1.85 (0.06)	1.85 (0.05)	1.92 (0.05)	1.84 (0.05)	1.95 (0.06)
Finger Temperature (°C)	32.38 (0.49)	31.70 (0.54)	32.28 (0.42)	32.14 (0.32)	32.16 (0.46)	31.88 (0.43)

**Table 3:** Mean arterial pressures (MAP) values at Baseline, the beginning, and end of RF-EMF exposure for the Sham, Low, and High Exposure conditions (standard errors in parentheses).

Time Point (min)	Sham			Low			High		
	Baseline	0–4	23–27	Baseline	0–4	23–27	Baseline	0–4	23–27
MAP	85.83 (9.22)	84.49 (7.69)	87.10 (8.03)	82.86 (6.27)	83.44 (6.30)	84.58 (6.85)	84.92 (7.98)	84.35 (7.42)	85.64 (8.69)
