# Correction: Loughran et al. Radiofrequency Electromagnetic Field Exposure and the Resting EEG: Exploring the Thermal Mechanism Hypothesis. *Int. J. Environ. Res. Public Health* 2019, *16*, 1505

**DOI:** 10.3390/ijerph23020157

**Published:** 2026-01-27

**Authors:** Sarah P. Loughran, Adam Verrender, Anna Dalecki, Catriona A. Burdon, Kyoko Tagami, Joonhee Park, Nigel A. S. Taylor, Rodney J. Croft

**Affiliations:** 1 Australian Centre for Electromagnetic Bioeffects Research (ACEBR), Illawarra Health and Medical Research Institute, School of Psychology, University of Wollongong, Northfields Ave, Wollongong, NSW 2522, Australia; adamv@uow.edu.au (A.V.); nigelastaylor@gmail.com (N.A.S.T.); rcroft@uow.edu.au (R.J.C.); 2 Centre for Population Health Research on Electromagnetic Energy (PRESEE), School of Public Health and Preventive Medicine, Monash University, Melbourne, VIC 3004, Australia; 3 Centre for Human and Applied Physiology, School of Medicine, University of Wollongong, Wollongong, NSW 2522, Australia

The authors have requested that the following changes be made to the original publication [1]. Upon further interrogation of the dataset from which our original publication was based, we have identified an error. Specifically, an in-house program that derived normalized values of ‘alpha’ electroencephalograph power had an error that notably affected the resultant alpha power values (and which were used statistically). That is, in the original article, it was reported that, relative to the sham condition, alpha power was similar during 1 W/kg RF-EMF exposure (*p* = 0.31), and significantly larger during 2 W/kg RF-EMF exposure (*p* = 0.02), whereas, after correcting the program, neither of these comparisons are statistically significant (*p* > 0.40). To account for this error, Figure 2 and the alpha power statistical results (Section 3.2) from the manuscript have been corrected, and all references to alpha power in the Abstract, Discussion and Conclusions sections have been amended to match the corrected results. This error did not affect any other physiological measures reported in the article (which are what the manuscript’s primary research question relates to). Accordingly, the demonstration that RF-EMF engages the thermoregulatory system, as well as our interpretation of this outcome, are not affected by the above error and thus remain valid.

In the Abstract, the sentences “Consistent with previous research, alpha EEG activity increased during the High exposure condition compared to the Sham condition. As a measure of thermoregulatory activation, finger temperature was found to be higher during both exposure conditions compared to the Sham condition, indicating for the first time that the effect on the EEG is accompanied by thermoregulatory changes and suggesting that the effect of RF-EMF on the EEG is consistent with a thermal mechanism.” have been changed to “Alpha EEG activity did not change during either of the exposure conditions compared to the Sham condition. As a measure of thermoregulatory activation, finger temperature was found to be higher during both exposure conditions compared to the Sham condition, indicating for the first time that RF-EMF exposure studies cause thermoregulatory changes. This supports the feasibility of the hypothesis that RF-EMF effects on alpha EEG activity are mediated via a thermal mechanism.”

Section 3.2, paragraph 1 should read as follows:

Resting EEG alpha power did not differ between the High RF-EMF exposure condition compared to the Sham condition (*p* = 0.43), and no difference was found between the Sham and Low RF-EMF exposure conditions (*p* = 0.40; see Figure 2 and Table 2).

Figure 2 should read:

Section 4: Discussion should read as follows:

Employing a tightly controlled methodology, the present study was designed to extend previous reports of RF-EMF exposure-related enhancements to the alpha band of the EEG and to determine, for the first time, the possibility that these alpha changes may be related to thermal effects on the central nervous system. The results did not replicate previous evidence for an impact of RF-EMF exposure on the alpha band of the EEG. However, the study demonstrated an increase in finger temperature in the exposure conditions relative to the Sham condition, which shows that the small but highly specific and localized thermal change caused by the RF-EMF exposure was sufficient to engage thermoregulatory processes.

Failing to replicate the majority of previous research, there was no increase in EEG alpha activity during RF-EMF exposure when compared to the Sham condition. Although previous studies have shown an impact on the EEG at lower exposure levels, similar to the 1 W/kg employed in our Low exposure condition (e.g., [5,30]), as well as some indication of a possible dose–response relationship (e.g., [31]), neither of those effects were observed in the current study. Furthermore, as detailed in Verrender et al. [21], no clear functional consequences of this change in EEG alpha activity were shown, providing further evidence that small changes in EEG activity do not have important functional consequences.

Novel to this experiment was the use of numerous physiological techniques to more tightly control (clamp) the heat content of the cutaneous tissues [32], and thereby remove artifactual thermal influences and stabilize skin temperatures. Importantly, all of the measures used were shown to be effective, verifying the utility of these methods. This is particularly important as the temperature changes accompanying RF-EMF-induced molecular oscillations are far smaller than those associated with local ambient influences. By minimizing those thermal ‘artifacts’, we were able to provide an insight into the mechanism underlying previously reported EEG alpha effects by tracking thermal physiological responses.

Despite the presence of whole-body clamping, finger temperatures were consistently higher during each of the RF-EMF exposures relative to the Sham condition. Since cutaneous blood flow to the hands and feet is exquisitely sensitive to changes in heat storage [33], the effect of which is most powerfully driven by thermosensitive neurons within the central nervous system [19], this would suggest that the observed increases in finger temperature, as a surrogate for local blood flow, reflected a physiological response (cutaneous vasodilation of the hands) to the RF-EMF-induced heating of the central thermosensitive tissues. The gradual reduction in finger temperature observed during each condition would be expected, since the participants remained stationary for an extended duration. Thus, the effect of RF-EMF-induced heating was to reduce the rate of that decline. As a consequence, this investigation may be the first to show a thermal mechanism that might underly the previously reported EEG alpha change.

Aside from the ramifications of the changes in finger temperature, the time course of those changes provides an interesting insight into how the effect might occur and in relation to the dose response of the effect. Specifically, the time course of the finger temperature response differed between the two exposure conditions, with an immediate thermoregulatory response observed in the High exposure condition and a weaker, more protracted effect seen in the Low exposure condition. Both exposures showed a similar effect on temperature by the end of exposure. This is an important consideration because, if the EEG effect is a result of thermal changes, then it would be expected that the EEG changes would also follow a similar time course, which is something that has not previously been taken into consideration within the RF-EMF EEG studies reporting on dose–response effects (e.g., [31]).

There are several important factors that require further consideration, both in the current study and for future studies. Firstly, since finger temperature is a surrogate for the local vascular responses, at least in the presence of thermal clamping, then it would seem that these RF-EMF exposures resulted in relatively inconsequential, but physiologically effective, increases in the temperature of the central thermosensitive neurons, the result of which appeared to be cutaneous vasodilatation. This underscores the importance of precisely regulating the thermal environment. Secondly, although the finger temperature data were consistent with a change in local blood flow, that observation requires verification. Thirdly, if the change in EEG alpha is a result of thermal changes as the present study might indicate, then equivalent temperature loads from non-RF-EMF sources should induce similar effects, although this is yet to be determined. Finally, our results imply that different levels of exposure can indeed result in different temporal patterns of thermal response. This has not previously been considered, and if EEG alpha changes are a result of a thermal response, this may help to explain the differences in effects reported by earlier studies, including less than reliable dose–response relations.

Section 5: Conclusions should read as follows:

The present study did not replicate previous evidence that the alpha band of the resting EEG is enhanced by RF-EMF exposures, commensurate with those emitted by modern telecommunications technologies. However, this is the first study to show that RF-EMF exposures are sufficient to engage a thermoregulatory response, and therefore it is consistent with an underlying thermal mechanism being responsible for the changes observed in brain physiology.

The authors apologize for any inconvenience. The authors state that the scientific conclusions are unaffected. This correction was approved by the Academic Editor. The original publication has also been updated.

## Figures and Tables

**Figure 2:**
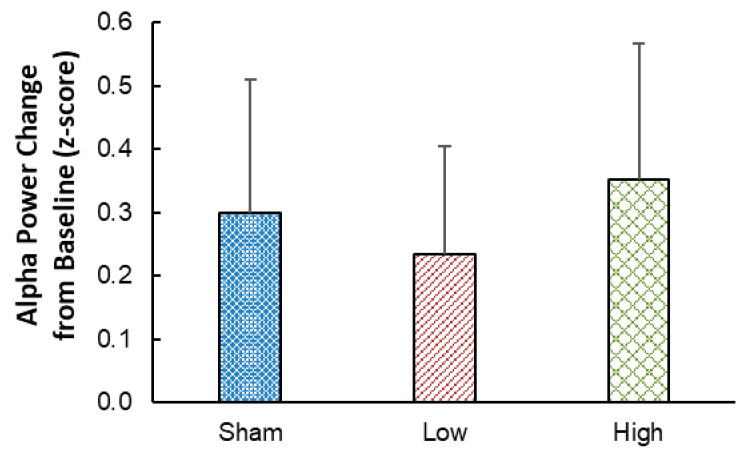
The change in EEG alpha power between Baseline and the end of exposure (min 22–26) in each of the Sham, Low, and High RF-EMF exposure conditions. Error bars denote standard errors of the means.

## References

[1] Loughran S.P., Verrender A., Dalecki A., Burdon C.A., Tagami K., Park J., Taylor N.A.S., Croft R.J. (2019). Radiofrequency Electromagnetic Field Exposure and the Resting EEG: Exploring the Thermal Mechanism Hypothesis. Int. J. Environ. Res. Public Health.

